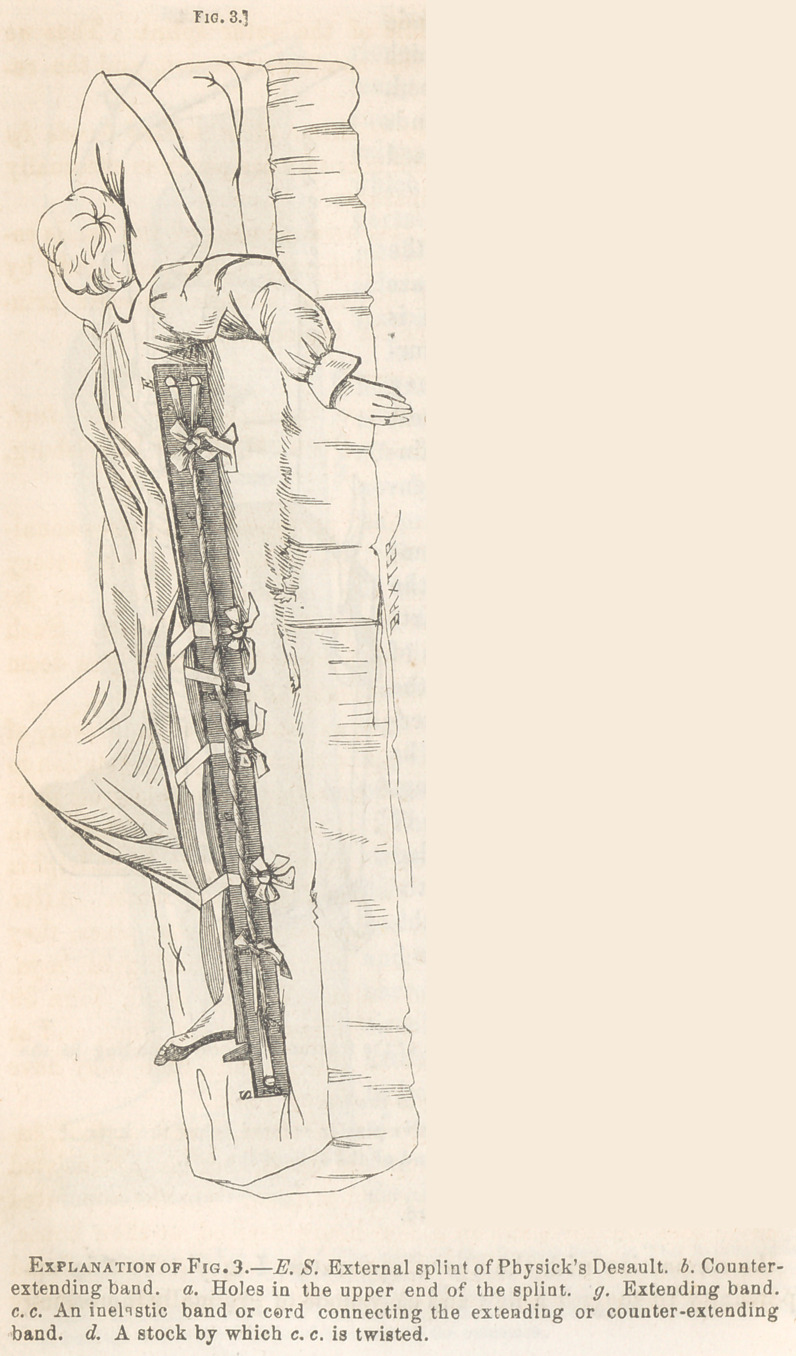# A New Apparatus for Fractures of the Leg, Requiring Extension and Counter-Extension

**Published:** 1855-10

**Authors:** John Neill

**Affiliations:** Professor of Surgery in the Pennsylvania College, Surgeon to the Pennsylvania and Philadelphia Hospitals


					﻿THE
MEDICAL EXAMINER.
NEW SERIES.—NO. CXXX.—0 C TO BE R, 1 8 5 5.
ORIGINAL COMMUNICATIONS.
A new Apparatus for Fractures of the Leg, requiring Extension
and Counter-Extension. By John Neill, M. D., Professor of
Surgery in the Pennsylvania College, Surgeon to the Pennsyl-
vania and Philadelphia Hospitals.
Comparatively few cases of fracture of the leg require the
employment of any apparatus by which extension and counter-
extension are effected. Simple means generally are sufficient in
ordinary cases.
There are, however, some cases of simple fracture involving
both bones, where the violence has been great, and where the line
of fracture is very oblique, or near the ankle, in which it is im-
possible to maintain the fragments in accurate adaptation without
resort to some means effecting permanent extension.
To accomplish this end various mechanical expedients are
employed with results equally variable. The double inclined
plane and its various modifications, Hutchinson’s splints, the
ordinary fracture-box, with the addition of the long splint of
Desault for fracture of the thigh, together with many numerous
patented machines, are the contrivances generally used.
I need only appeal to the expe-
rience of those under whose care
many such cases occur, to the nume-
rous deformities in every extensive
cabinet, and to the great ingenuity
which has been displayed in this de-
partment of mechanical surgery, to
establish the truth that there is a de-
sideratum in the treatment of this
fracture.
In endeavoring to supply this de-
ficiency, I have resorted to a con-
trivance which I think is simple and
effective, and which I believe to be
novel, not only in its details, but in
its principle.
For simple fractures of both bones
of the leg, attended with shortening
and deformity not easily overcome,
the limb should be placed in a long
fracture-box, with sides extending as
high as the middle of the thigh, and
a pillow should be used for com-
presses.
The counter-extension is made by
strips of adhesive plaster one and a
half inches in breadth, secured on
each side of the leg below the knee
and above the seat of fracture, by
narrower strips of plaster applied
circularly. The end of the counter-
extending strips may then be secured
to holes in the upper end of the sides
of the fracture-box, by which the
line of the counter-extension is ren-
dered nearly parallel with the limb.
In connection with the above apparatus, I may take this op-
portunity to suggest a new and easy mode of gradually increasing
The extension is also to be made
by adhesive strips in a mode which
is now well known and understood.
The ends of the extending bands
may be fastened to the foot-board
of the box.
In compound fractures of the
leg, shortening and deformity are
often difficult to overcome, as is
well known to experienced sur-
geons. In such cases we may
wish to dress the wounded soft
parts, and at the same time main-
tain a certain amount of exten-
sion and counter-extension.
This can be readily accom-
plished by having the sides of the
fracture-box sawed in two parts
at the knee, so that the sides of
the box above the knee, from the
upper ends of wffiich the counter-
extension is made, need not be
disturbed during the dressing,
while that portion of the side of
the box corresponding to the leg
may be opened at pleasure, with-
out diminishing the tension of the
extending or counter-extending
bands.
the tension force in machines
which may be applied either
to fractures of the thigh or
to the leg.
The object is often ob-
tained by simply tying the
ends of the extending and
counter-extending bands to
the extremities of the splint,
or where it is found difficult
to maintain the desired de-
gree of tension, by screws
and ratchets of various forms
applied to the ordinary
splints. The improvement
consists in making the ex-
tension and counter-extension
in a continuous line, and of a
simple means of gradually
and powerfully increasing the
force.
To illustrate this applica-
tion, take, for instance, a
case of fracture of the thigh,
in which a Physick’s Desault
has been applied in the usual
way. Instead of securing
the extremities of the counter-
extending perineal band to
the two holes in the upper
end of the splint, and the
extremities of the extending
band to the hole in the lower
end of the splint, let these
bands be carried through their
respective holes and secured
to each other about the middle of the outer splint. Thus we
make the extension increase the counter-extension, and the re-
verse.
At the same time, by simply twisting these united bands by
a small stick, we can increase and maintain the power as effectually
as by the most complicated apparatus.
In simplicity and in power, this arrangement of the bands re-
sembles the Spanish windlass or temporary tourniquet, made by
a handkerchief and stick. Its operation is upon the same prin-
ciple as Gilbert’s Twisted Rope for Dislocation.
				

## Figures and Tables

**Fig. 1. f1:**
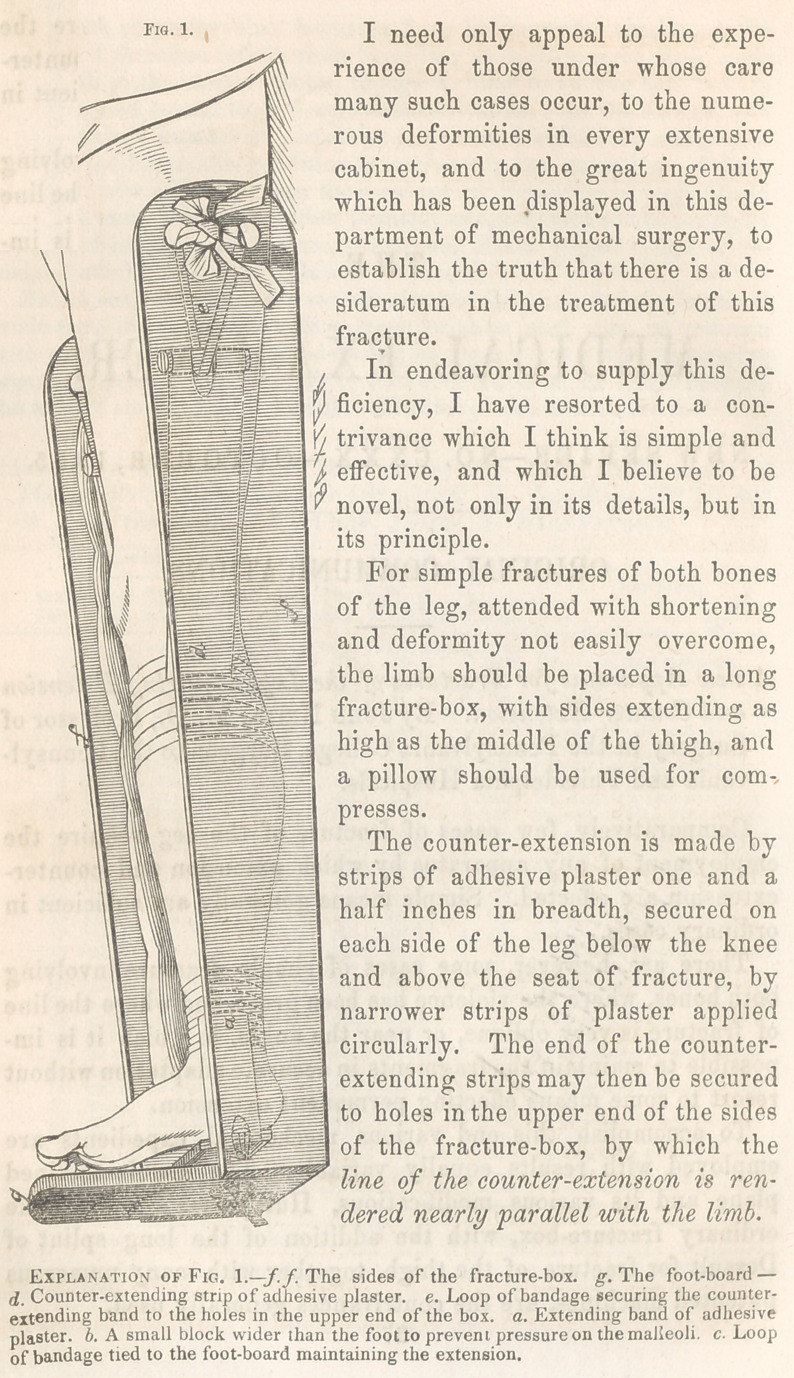


**Fig. 2. f2:**
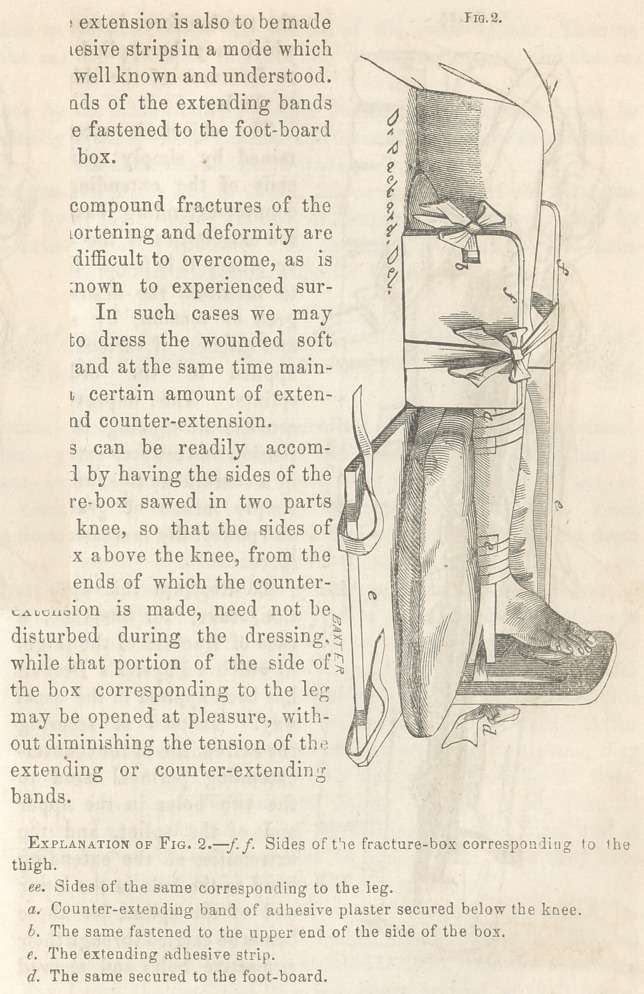


**Fig. 3. f3:**